# A multi-layered disruption: how calcium, acidification, and ROS signals converge to shut down energy metabolism in incompatible pollen

**DOI:** 10.1093/plcell/koag046

**Published:** 2026-02-21

**Authors:** Ved Prakash

**Affiliations:** Assistant Features Editor, The Plant Cell, American Society of Plant Biologists; Department of Plant Pathology, The Ohio State University, Wooster, OH 44691, United States

Self-incompatibility (SI) is a pollen-pistil recognition system that prevents inbreeding by inhibiting self-pollen fertilization, thereby promoting outcrosses and increasing genetic variation. The molecular mechanisms underlying SI vary significantly across different plant families (Kao and Tsukamoto 2004).

In *Papaver rhoeas* (poppy), during an SI interaction the growing pollen tube undergoes a multi-layered chain of events that rapidly leads from a high-energy growth state to growth inhibition followed by a state of severe metabolic disruption. This transition is driven by a calcium-dependent signaling network, starting with a cytosolic calcium concentration increase, reactive oxygen species (ROS) formation, and cytosolic acidification. These events are accompanied by rapid alterations in mitochondrial morphology and rapid ATP depletion (Geitmann et al 2004; Wang et al 2022). Programmed cell death of incompatible pollen finally occurs several hours later, as a downstream consequence of this earlier signaling cascade (Bosch and Franklin-Tong 2024). However, precise information regarding the subcellular sources and targets of ROS generation, the broader implications of cytosolic calcium accumulation and acidification, and the underlying mechanisms driving rapid ATP depletion in pollen during SI remain scarce.

Recent work by Ludi Wang and colleagues (Wang et al 2026) uses *Papaver* and a heterologous system in *Arabidopsis thaliana* (expressing *Papaver* SI proteins) to decipher an interlinked SI signaling network: cytoplasmic calcium, pH, H_2_O_2_, and mitochondria converge to collapse energy generation in pollen tubes (see Fig. 1). Spatial distribution of ROS can be experimentally assessed: nitroblue tetrazolium staining is widely used to measure apoplastic superoxide, while roGFP2-Orp1 (a genetically encoded H_2_O_2_ sensor) can be exploited to monitor H_2_O_2_ within specific subcellular compartments. Using nitroblue tetrazolium staining, they report that SI inhibits pollen tube tip-localized plasma membrane NADPH oxidase activity, which rapidly disrupts apoplastic superoxide production toward the pollen tube tip. To identify relevant intracellular sources of ROS production during SI, they exploited the roGFP2-Orp1 sensor expression in the Arabidopsis heterologous system. Results reveal a SI-induced H_2_O_2_ production in cytosol, mitochondria, and plastids but not in peroxisome or nucleus. Interestingly, following SI induction, the authors detected H_2_O_2_ in cytosol and mitochondria as early as 10 min, while in plastids after 5 min, suggesting that SI-induced ROS production is a rapid response.

**Figure 1:**
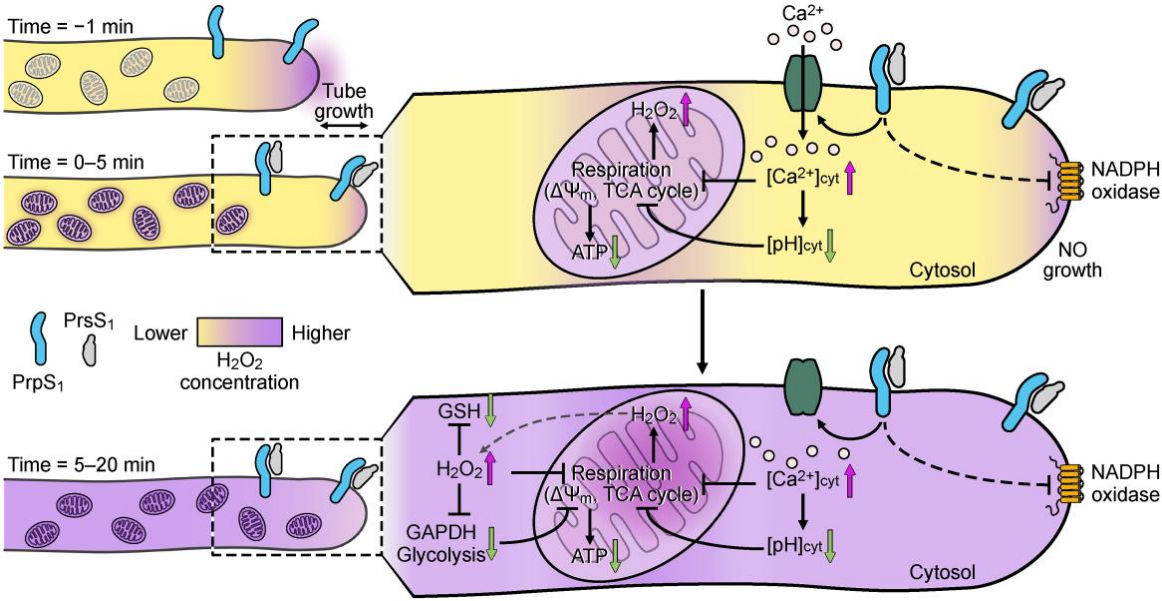
**Proposed model for SI signaling network during the first 10 to 20 min in incompatible pollen tubes.** Upon interaction of the *Papaver* pollen S-determinant (PrpS1) with the self-pistil ligand (PrsS1), calcium influx and acidification trigger mitochondrial dysfunction, leading to H_2_O_2_ production, reduced levels of antioxidants (GSH), and GAPDH inactivation. This cascade causes metabolic collapse and ATP depletion, ultimately leading to intracellular disruption. Reprinted from Wang et al. (2026), Fig. 10.

Mitochondrial electron transport chain (ETC) generates H_2_O_2_ as a byproduct of respiration. To investigate the role of ETC into H_2_O_2_ generation during SI, Wang and colleagues performed an antimycin A–induced pharmacological inhibition assay of the complex III of ETC. Interestingly, antimycin A disrupted the SI-induced increases in roGFP2-Orp1 oxidation in mitochondria and the cytosol, suggesting ETC is a contributor to the SI-induced H_2_O_2_ production in these compartments.

Building on earlier research that showed how “self” recognition causes pollen to run out of energy (Wang et al 2022), the authors now explain exactly how mitochondrial dysfunction causes this rapid power loss. H_2_O_2_ treatment of the pollen tubes showed rapid depletion of ATP level, suggesting that H_2_O_2_ directly affects energy production. Since ATP production by ATP synthase depends on mitochondrial membrane potential (Δ *ψ* m), the authors also investigated Δ *ψ* m in SI. They demonstrated that both SI and H_2_O_2_ induced Δ *ψ* m collapse leading to rapid ATP depletion. As this is not the cause of pollen tube arrest, which happens earlier, they investigated what other changes are triggered in incompatible pollen. Using cytoplasmic calcium indicator Yellow Cameleon 3.6 and tetramethylrhodamine methyl ester probe, the authors simultaneously imaged cytosolic calcium and Δ *ψ* m and found that the elevated cytoplasmic calcium is an upstream event of Δ *ψ* m collapse during SI. In addition to calcium, cytosolic acidification plays role in SI-induced mitochondrial dysfunction and ROS generation. One of their major findings is that both SI and H_2_O_2_ negatively affect glycolysis, Krebs cycle, and redox homeostasis in *Papaver* pollen tubes (see Fig. 1).

In conclusion, the work of Wang and colleagues provides the details of the molecular mechanisms underlying rapid disruption of pollen tube energy metabolism during SI. They report a mitochondria-centered interconnected signaling network in which the interplay of calcium, ROS, and pH acts as an extremely efficient biological mechanism to dismantle the cell's energy infrastructure rather than simply inhibiting growth. These findings provide a new mechanistic framework for understanding how plants govern pollen compatibility and promote genomic diversity.

## Recent related articles in *The Plant Cell*:

- Tang et al. (2023) demonstrated the molecular mechanism of pollen tube swelling during SI in pear.
- Wang et al. (2024) reported a step-by-step process of PCD using *Arabidopsis* root tip.
- Mabry et al. (2024) proposed using *Arabidopsis* resources to establish the entire Brassicales order as a “model clade” for studying plant evolution.

## Data Availability

None to declare.

## Dive Curated Terms

The following phenotypic, genotypic, and functional terms are of significance to the work described in this paper:

ATP CHEBI: CHEBI:15422

NADPH CHEBI: CHEBI:16474

self-incompatibility Planteome: TO:0000310
